# Efficacy of citric acid denture cleanser on the *Candida albicans* biofilm formed on poly(methyl methacrylate): effects on residual biofilm and recolonization process

**DOI:** 10.1186/1472-6831-14-77

**Published:** 2014-06-23

**Authors:** Fernanda Faot, Yuri Wanderley Cavalcanti, Martinna de Mendonça e Bertolini, Luciana de Rezende Pinto, Wander José da Silva, Altair Antoninha Del Bel Cury

**Affiliations:** 1 Department of Prosthodontics, School of Dentistry, Federal University of Pelotas, Pelotas, Rio Grande, do Sul 96015-560, Brazil; 2 Department of Prosthodontics and Periodontology, Piracicaba Dental School, State University of Campinas, Piracicaba, São Paulo 13414-903, Brazil

**Keywords:** Biofilm, Denture hygiene, Denture Cleansers, *Candida albicans*, Poly(methyl methacrylate)

## Abstract

**Background:**

It is well known that the use of denture cleansers can reduce *Candida albicans* biofilm accumulation; however, the efficacy of citric acid denture cleansers is uncertain. In addition, the long-term efficacy of this denture cleanser is not well established, and their effect on residual biofilms is unknown. This *in vitro* study evaluated the efficacy of citric acid denture cleanser treatment on *C. albicans* biofilm recolonization on poly(methyl methacrylate) (PMMA) surface.

**Methods:**

*C. albicans* biofilms were developed for 72 h on PMMA resin specimens (n = 168), which were randomly assigned to 1 of 3 cleansing treatments (CTs) overnight (8 h). CTs included purified water as a control (CTC) and two experimental groups that used either a 1:5 dilution of citric acid denture cleanser (CT5) or a 1:8 dilution of citric acid denture cleanser (CT8). Residual biofilms adhering to the specimens were collected and quantified at two time points: immediately after CTs (ICT) and after cleaning and residual biofilm recolonization (RT). Residual biofilms were analyzed by quantifying the viable cells (CFU/mL), and biofilm architecture was evaluated by confocal laser scanning microscopy (CLSM) and scanning electron microscopy (SEM). Denture cleanser treatments and evaluation periods were considered study factors. Data were analyzed using two-way ANOVA and Tukey’s Honestly Significant Difference (HSD) test (α = 0.05).

**Results:**

Immediately after treatments, citric acid denture cleansing solutions (CT5 and CT8) reduced the number of viable cells as compared with the control (p < 0.01). However, after 48 h, both CT groups (CT5 and CT8) showed biofilm recolonization (p < 0.01). Residual biofilm recolonization was also detected by CLSM and SEM analysis, which revealed a higher biomass and average biofilm thickness for the CT8 group (p < 0.01).

**Conclusion:**

Citric acid denture cleansers can reduce *C. albicans* biofilm accumulation and cell viability. However, this CT did not prevent biofilm recolonization.

## Background

*Candida* spp. is one of the main causative organisms of denture-induced stomatitis, which is primarily due to its ability to adhere and form biofilms on oral cavity tissues and denture surfaces, as well as due to its resistance to antifungal agents [1-4]. This biofilm grows extensively on acrylic resin denture material and its effective removal is a significant challenge by both chemical and mechanical methods [2-5].

Many chemical denture cleansers that contain enzymes, sodium hypochlorite, alkaline peroxide, and acid solutions are available for use with mechanical brushing to remove the residual biofilm attached to denture surfaces [5-8]. Denture cleansing solutions have antimicrobial properties, as demonstrated by many studies [7-12]; however, none of these methods seem to effectively remove the biofilm and prevent recolonization on the denture surface [6,7].

Another type of denture cleanser contains citric acid and is available as a concentrated solution, which can be used daily (1:5 dilution) or weekly (1:8 dilution) after proper dilution (as indicated by the manufacturer). This cleanser acts as a chemotherapeutic agent that can effectively disrupt biofilms through a sequestering mechanism with calcium ions [13]. This mechanism allows citric acid to break calcium bridges and subsequently disrupt the biofilm matrix, which may lead to anti-biofilm activity [14,15].

Citric acid solutions were evaluated for their ability to decontaminate implant surfaces [16], demonstrating a reduction in the number of pathogenic species. Although citric acid solutions are also effective against *Streptococcus mutans* biofilms and biofilms derived from multiple species that develop on titanium surfaces [16], the effect of citric acid cleansers against *Candida* biofilms on denture surfaces has not yet been reported.

Although continuous use of citric acid cleansers for 3 months showed adverse effects with greater ion release from Co–Cr alloys [17,18], no harmful effects have been demonstrated with denture materials in general [19]. In contrast, other denture cleansers, mainly those containing sodium hypochlorite, could increase surface roughness, decrease hardness, and eventually change the color of acrylic resins and denture liners [19-21]. Therefore, citric acid cleansers might be suitable for removable dentures and orthodontic appliances and for removing biofilms and preventing their recolonization.

Therefore, considering the scarcity of studies examining the efficacy of citric acid cleansers for removing biofilms from denture materials, the present study evaluated the efficacy of citric acid cleansers on *Candida albicans* biofilm recolonization on poly(methyl methacrylate) (PMMA).

## Methods

### Experimental design

This *in vitro* study used a randomized, blinded design. *C. albicans* biofilms were developed for 72 h on PMMA resin specimens (n = 168) and then randomly assigned to 1 of 3 cleansing treatments (CTs): purified water, used as a control (CTC); 1:5 dilution of citric acid denture cleanser (CT5); or 1:8 dilution of citric acid denture cleanser (CT8). Residual biofilms adhering to specimens were collected and quantified at two different time points: immediately after cleaning treatments (ICT group) or 48 h after the cleaning, when residual biofilm recolonization (RT group) would occur. Residual biofilms were analyzed by determining the number of viable cells (CFU/mL). Biofilm architecture was evaluated by confocal laser scanning microscopy (CLSM) and scanning electron microscopy (SEM). The study factors were denture cleanser treatments and evaluation periods (ICT or RT). The response variables were the number of viable cells and the architecture of *C. albicans* residual biofilms. A scheme of the experimental design is illustrated in the Additional file 1.

### Resin specimens

Disc-shaped specimens (10 mm diameter, 2 mm thickness, and 219.8 mm^2^ area) of microwave-polymerized PMMA (Onda Cryl; Artigos Odontológicos Clássico Ltd, São Paulo, Brazil) were fabricated according to the manufacturer’s instructions. After polymerization, the discs were immersed in purified water at 37°C for 48 h for residual monomer release [22]. The PMMA specimens were then ground with a horizontal polisher (model APL-4; Arotec, São Paulo, Brazil) and using progressively finer aluminum oxide papers (320-, 400-, and 600-grit) to standardize surface roughness at 0.34 ± 0.02 μm. Before developing the biofilm, the discs were ultrasonically cleaned (Thornton T 740; Thornton-Inpec Eletrônica Ltda, Vinhedo, Brazil) with 70% alcohol and sterilized ultra-purified water (20 min) to remove contaminants and artifacts from the surface [23]. The absence of contamination was confirmed by immersing a sample of the specimens in sterile culture media.

The specimens were randomly assigned to 1 of the 3 treatment groups, which were further divided within the evaluated time points: immediately after treatments (ICT) and 48 h after cleaning and residual biofilm recolonization (RT). The number of specimens in each group (n = 12) was determined with preliminary tests, which confirmed the sample size yielded an adequate power (80%) for detecting statistically significant differences.

### Salivary pellicle formation on specimens

Before developing the biofilm assays, clean PMMA specimens were coated with saliva to mimic the oral cavity environment. Human whole saliva was donated by a healthy volunteer, who provided written informed consent. The Research and Ethics Committee of Piracicaba Dental School, State University of Campinas, approved this study. The saliva sample was collected at the same time of day, for each experiment, and the collection volume was limited to 50 mL per collection period.

Human whole saliva was collected by masticatory stimulation with flexible film (Parafilm M; American Can Co, Neenah, WI). Saliva was clarified by centrifugation at 3,800 *g* for 10 min at 4°C. The supernatant was collected and sterilized by filtration (22 μm) for immediate use. For each disc, a salivary pellicle was formed on the surface after incubation for 30 min, at 37°C and 75 rpm, in an orbital shaker.

### Inoculum and growth conditions

A loopful of yeast culture of *C. albicans* (ATCC 90028) was reactivated and incubated for 24 h at 37°C. Afterwards, cells were harvested, suspended in Yeast Nitrogen Base (YNB) broth (Becton Dickinson, Franklin Lakes, NJ) supplemented with 100 mM glucose. Cell density was spectrophotometrically (Spectronic 20; Bausch & Lomb, Rochester, NY) standardized to a 0.25 optical density at 520 ηm, which corresponded to 1 × 10^6^ CFU/mL inoculum [24].

### Biofilm assay

PMMA saliva-coated discs were placed vertically in polystyrene 24-well culture plates. Subsequently, 2 mL of the standardized cell suspension (1 × 10^6^ CFU/mL of *C. albicans* in YNB supplemented with glucose 100 mM) was added to each well. Biofilm was developed at 37°C, and under 75 rpm in an orbital shaker, for 72 h, to allow biofilm maturation. The medium was changed every 24 h. Biofilm assays were performed in triplicate in 3 independent experiments.

### Treatment protocols

After the biofilm growth for 72 h, the specimens were randomly assigned to 1 of 3 CTs overnight (8 h). Citric acid cleanser (CURADEN BDC 105, Curaprox, Swiss) was diluted in purified water, according to the manufacturer’s instructions, in 1:5 or 1:8 solutions, which are recommended for weekly or daily use, respectively. Each specimen was placed in a sterile beaker containing 8 mL of the treatment solution.

Following cleanser treatments (8 h), specimens were removed and washed twice in 2 mL of sterilized phosphate-buffered saline (PBS) solution (pH 7.4). The residual biofilms adhering to the specimens were immediately collected (ICT) or allowed to grow under the same conditions for 48 h (RT group) [10].

### Viable cell quantification from residual biofilm

Residual biofilms were disrupted and adhered microorganisms were removed from specimens by sonication (7 W for 30 s). Sonicated solutions were serially diluted in PBS and plated (20 μL) in triplicate on Sabouraud Dextrose agar. The plates were incubated at 37°C under aerobic conditions for 48 h. CFU were counted using a stereomicroscope, and the results are expressed in colony-forming units per mL (CFU/mL).

### Biofilm architecture analysis: SEM

Specimens with attached biofilms were rinsed with sterile PBS and placed in 1% osmium tetroxide for 1 h. Specimens were subsequently washed in purified water, dehydrated in a series of ethanol washes (70% for 10 min, 95% for 10 min, and 100% for 20 min) and air-dried in a desiccator before sputter coating with gold [24,25]. Next, specimens were mounted on aluminum stubs and coated with gold. The biofilm surface features were visualized with SEM (JSM 5600LV, JEOL, Tokyo, Japan) at 1,500× in a high-vacuum mode at 15 kV.

### Biofilm architecture analysis: CLSM

Biofilms formed on PMMA surfaces were stained using the Live/Dead BacLight Viability kit, comprising SYTO-9 and propidium iodide (PI). Before the CLSM examinations, specimens were protected from light and incubated at 37°C for 20 min [24,25]. Images of stained biofilms were captured using a CLSM system (LEICA – TCS SP5, Leica Microsystems, Wetzlar, Germany). A series of images were obtained at 1-μm intervals in the z section for a three-dimensional view of the biofilm. At least, five representative optical fields were examined for each specimen.

COMSTAT software was used to analyze CLSM images. The architecture properties of biofilms analyzed by COMSTAT included the biovolume (μm^3^/μm^2^), average thickness (μm), and roughness coefficient.

### Statistical analysis

Data were analyzed using statistical software (SAS v. 9.0; SAS Institute, Inc, Cary, NC) with a significance level fixed at 5%. The assumptions of equality of variances and normal distribution of errors were evaluated for each variable. When normality was violated, the data were logarithmically transformed. Factors interfering in the response variables (*C. albicans* viable cells, CFU/mL), bio-volume (μm3/μm2), average thickness (μm) and roughness coefficient) were analyzed with two-way ANOVA (type and evaluation period). Post-hoc comparisons were performed using Tukey’s Honestly Significant Difference (HSD) test.

## Results

The two-way ANOVA comparisons showed that both study factors “treatments” (CTC, CT5, and CT8) and “evaluation periods” (ICT and RT) affected the viable cell quantification (p < 0.01); but no statistical interactions were detected. However, for biofilm architecture parameters (bio-volume, average thickness, and roughness), the two-way ANOVA comparisons showed a statistical significant interaction (p < 0.001) between the factors under study.

The use of citric acid cleansers resulted in no viable cell counts immediately after treatments for both experimental groups (CT5 and CT8) (p < 0.01), as showed on Table 1. However, 48 h after treatments, *C. albicans* CFU counts were detected in the viable cell quantification, demonstrating that residual biofilms could recolonize the PMMA surface specimens. Although citric acid CTs were not effective within 48 h (p < 0.01), these cleaning solutions showed lower number of CFU counts when compared with the control group (p < 0.01) at both evaluation time points.

**Table 1:** ***C. albicans *** **viable cell quantification (average ± SD) according to different treatments and evaluation period**

	**Viable Cell Counts (10**^**6**^ **CFU/mL)**	
**Cleansing treatment**	**Evaluation periods**	
	**Immediately after treatments (ICT)**	**Allowed to recolonize (RT)**
CTC - H_2_O	1.01 ± 8.59^Aa^	11.1 ± 12.30^Ba^
CT5 - 1:5	0^Ab^	1.92 ± 3.64^Bb^
CT8 - 1:8	0^Ab^	3.63 ± 4.12^Bb^

Different uppercase letters indicate statistically significant differences (p < 0.01) between the evaluation periods [Immediately treated (ICT) and allowed to recolonize (RT)]. Distinct lowercase letters indicate statistically significant differences (p < 0.01) among treatments (CTC, CT5, and CT8).

**Figure 1:**
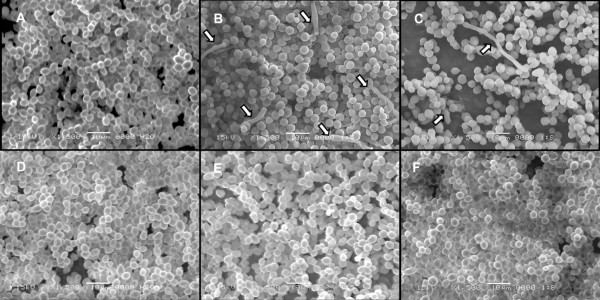
**Representative scanning electron microscopy (SEM) images (1,500×) of *C. albicans *biofilms according to different treatments and evaluation periods: A, CTC group (ICT); B, CT5 group (ICT); C, CT8 group (ICT); D, CTC group (RT); E, CT5 group (RT); F, CT8 group (RT).** Note the hyphal (arrows) formation immediately after both citric acid solution treatments in ICT and the recolonization after 48 h (RT).

**Figure 2:**
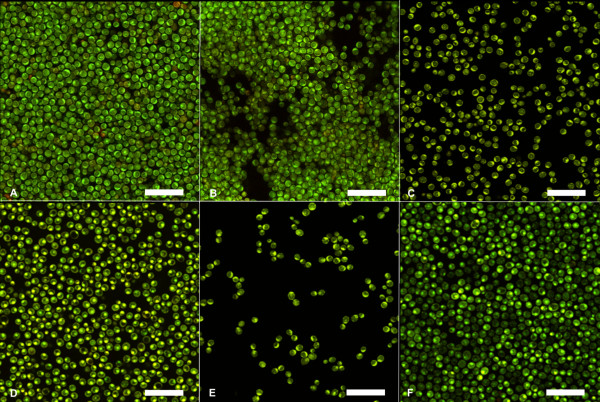
**Representative confocal laser scanning microscopy (CLSM) of *C. albicans *biofilms according to different treatments and evaluation periods in the evaluated treatments and periods: A, CTC group (ICT); B, CT5 group (ICT); C, CT8 group (ICT); D, CTC group (RT); E, CT5 group (RT); F, CT8 group (RT).** Bars represent 12.5 μm. Note lower cell density in ICT, for both CT5 and CT8 groups **(B and C)**, in comparison to control **(A)**. Note the lower cell density for the CT5 group compared with the CT8 group in RT. Slight reduction in cell density for the control group may be due to biofilm over-maturation.

Biofilm architecture analyses are presented in Table 2. Statistically significant differences were detected for evaluation periods (p < 0.01) and for denture cleanser treatments (p < 0.05). Immediately after treatments (ICT), biofilms treated with CT5 were more affected than those treated with CT8 and the control groups, showing a lower bio-volume and average thickness than those for the other groups (p < 0.05). In addition, the CT8 group showed a higher roughness coefficient, indicating the biofilm was less compacted (p < 0.05). After the recolonization period (RT), biofilms treated with daily cleansing solution (CT8) showed an increase in bio-volume and in average thickness when compared with those for the 1:5 dilution (CT5) and control (water) treatments (p < 0.05).Although there were no statistical differences in the architecture of biofilms treated with water or CT5 (p > 0.05), differences in biofilms visualized by SEM and CLSM were detected among groups (Figures 1 and 2). As the figures suggest, biofilms from the control and citric acid groups showed different metabolic levels after each CTs for both evaluation periods.Figure 1 presents representative SEM images of biofilms treated with purified water, CT5, and CT8 at the ICT and RT evaluation periods. Regarding the control treatment (purified water), no substantial changes in biofilm structure were detected. Hyphae were observed immediately after citric acid treatments (Figure 1B and C). We observed a dramatic reduction in the number of cells for the CT8 group immediately after treatments (Figure 1C). In addition, we observed a slight reduction in cell numbers for CT5 at both time points (Figure 1B and E), compared with the control. Biofilms were identified for all groups after the recolonization period (Figure 1D, E and F) and larger number of cells were observed for the CT8 group at RT (Figure 1F).

**Table 2:** **Bio-volume (μm**^**3**^**/μm**^**2**^**), average thickness (μm), and roughness coefficient (average ± SD) of *C. albicans *** **biofilms according to different treatments and evaluation period**

	**Bio-volume**		**Average thickness**		**Roughness coefficient**	
**Treatments**	**Evaluation periods**		**Evaluation periods**		**Evaluation periods**	
	**ICT**	**RT**	**ICT**	**RT**	**ICT**	**RT**
CTC	10.77 ± 1.5^Aa^	1.30 ± 1.2^Ba^	12.25 ± 2.1^Aa^	1.53 ± 1.4^Ba^	0.11 ± 0.1^Aa^	1.41 ± 0.4^Ba^
CT5	12.61 ± 3.1^Aa^	1.14 ± 0.6^Ba^	11.97 ± 3.1^Aa^	1.04 ± 0.6^Ba^	0.21 ± 0.2^Aa^	1.73 ± 0.1^Ba^
CT8	3.87 ± 2.8^Ab^	9.71 ± 2.7^Bb^	4.88 ± 3.7^Ab^	11.69 ± 3.3^Bb^	1.17 ± 0.3^Ab^	0.30 ± 0.2^Bb^

Different uppercase letters indicate statistically significant differences (p < 0.01) between the evaluation periods within treatments [Immediately treated (ICT); and Allowed to recolonize (RT)]. Distinct lowercase letters indicate statistically significant differences (p < 0.05) among treatments (CTC, CT5, and CT8) within periods of evaluation.

## Discussion

In the present study, we evaluated the efficacy of citric acid denture cleanser for removing or killing the *C. albicans* biofilm formed on the surface of PMMA specimens. A long-term effect of other denture cleansing solutions was demonstrated in previous studies [7,9], which also included evaluation of the gold standard solution (sodium hypochlorite). In the present study, only purified water was used as the control. Considering that it is well known that sodium hypochlorite is effective against *Candida* biofilms, our study aimed to show differences, if any, between citric acid denture cleanser and the absence of chemical treatment. The present study showed that the citric acid treatment was more effective than the absence of treatment; however comparisons with a gold standard solution remain to be tested.

The citric acid denture cleanser used in the present study was a blend of ultra-purified water and citric acid in non-toxic, water soluble, low-viscosity chemical solution, which can break calcium-ion bridges that serve as chemical binding sites connecting the EPS polymeric chains [13]. Based on the literature, citric acid is the chemotherapeutic agent with the highest potential for removing biofilms from contaminated titanium surfaces *in vitro*, although it did not achieve complete removal [14,16].

In the present study, we observed a biofilm recolonization phenomenon in both experimental groups when cleaned specimens were maintained for 48 h more in a culture medium supplemented with glucose. Therefore, we found that citric acid disrupted the biofilms but did not totally remove them. This finding leads to the supposition that this denture cleanser can be an effective complementary method for biofilm removal once it allows the debris to be more easily removed from the denture surface. Thus, it is expected that citric acid presents a more consistent effect on biofilm removal when associated with a mechanical method, such as brushing, to completely remove the mature biofilm from dentures [14,16,26].

Although the number of viable cells was null immediately after treatments with either citric acid cleansers, it should be noted that the number of viable cells identified by CFU assay has low sensibility when a small number of cells are viable. In an attempt to explain these results, we used CLSM as an auxiliary method for CFU assay by analyzing the biofilms with their three-dimensional structures preserved, which also showed viability. Therefore, the presence of residual biofilm was confirmed by SEM and CLSM analysis, and it was possible to observe reminiscent viable cells and biofilm structures in ICT. These residual biofilms contributed to surface recolonization after 48 h, as was observed in RT.

The present study evaluated the 1:5 and 1:8 dilutions of citric acid cleanser to simulate its weekly and daily use. These solutions probably did not completely affect the basal layers of the biofilms. This phenomenon may be due to the presence of an extracellular matrix, which protected the biofilm from the cleanser, and the reminiscent cells in metabolic quiescence, which accounted for the recolonization after 48 h [1,27]. Therefore, a single exposition to citric acid denture cleanser is not able to remove *Candida* biofilms, or prevent their recolonization.

Comparing the results from viable cell quantification and biofilm architecture analysis, we found that treatments with 1:5 and 1:8 dilutions of the citric acid solutions might have a delayed effect on the biofilm cells. Although many viable cells were seen in the CLSM images, these were not detected by viable cell assessment. This can be explained by the phenomenon known as Post-Antifungal Effect (PAFE), in which substances stored inside vacuoles might kill the cells over time [4].

According to PAFE, citric acid uptake by *Candida* cells during the treatments contributed to the absence of CFUs in the viable cell assessment, as well as the reduction in cell density for the 1:5 and 1:8 groups, as compared with the control. With regard to the biofilms visualized in RT, a higher biomass was observed for the CT8 group than for the CT5 group. This difference may be related to differences in the damage provoked by the two cleansers in RT, meaning that CT8 treatment allowed higher biofilm recolonization at the RT evaluation period.

According the PAFE and the results obtained with the CLSM images, biofilms treated in the 1:5 group showed greater damage than those in the 1:8 group. Basal layers from the biofilms treated with the 1:5 dilution seemed to show a greater response at ICT. This is because the 1.5 dilution treatment resulted in a lower biofilm bio-volume and average thickness at RT. Furthermore, the 1:8 dilution treatment did not deeply affect biofilms, allowing their extensively recolonization after 48 h.

The results of the present study demonstrate that citric acid denture cleanser is effective in reducing *C. albicans* cell viability in a mature biofilm, immediately after treatments. However, this cleansing solution does not completely remove the biofilm and does not prevent its recolonization after 48 h. Therefore, this study presents important findings regarding the anti-biofilm effect of citric acid denture cleanser.

The results of the present study should be interpreted with care because of the *in vitro* nature of the 72 h formed biofilm does not fully match the environment of the oral cavity. Also, during the initial 72 hours of initial biofilm formation, used to simulate a mature biofilm, there would be several microbial successions through time, which could be somewhat a limitation of the present study. However, the results provide important data on how the *C. albicans* biofilm behaves with daily and weekly treatments with citric acid denture cleanser used in clinical practice. Additional studies are needed with other *Candida* species in a single or mixed biofilm, which are increasingly implicated in long-term denture stomatitis. Also, these biofilms should be used for comparisons between the long-term efficacy of such treatments with citric acid and a gold standard solution (sodium hypochlorite), which would benefit the clinical treatment approaches.

## Conclusion

Within the limitations of this study, we found that citric acid denture cleansers reduced cell viability but did not prevent biofilm recolonization within 48 h.

## Competing interests

The authors declare that they have no competing interests.

## Authors’ contributions

FF, LRP, WJS, and AADBC conceptualized and designed the study. FF, LRP, and WJS collected the data. YWC and MMB interpreted and analyzed the data. FF, YWC, MMB, and LRP drafted the manuscript. WJS and AADBC performed the final review of the manuscript. All authors read and approved the final version of this manuscript.

## Pre-publication history

The pre-publication history for this paper can be accessed here:

http://www.biomedcentral.com/1472-6831/14/77/prepub

## Supplementary Material

Additional file 1 Scheme of the experimental design performed in the present study. Click here for file
