# Supplementary figures and images for: Efficacy of citric acid denture cleanser on the Candida albicans biofilm formed on poly(methyl methacrylate): effects on residual biofilm and recolonization process
Source: BMC Oral Health. 2014 Jun 23;14:77. doi: 10.1186/1472-6831-14-77 (PMC4076759; doi:10.1186/1472-6831-14-77)

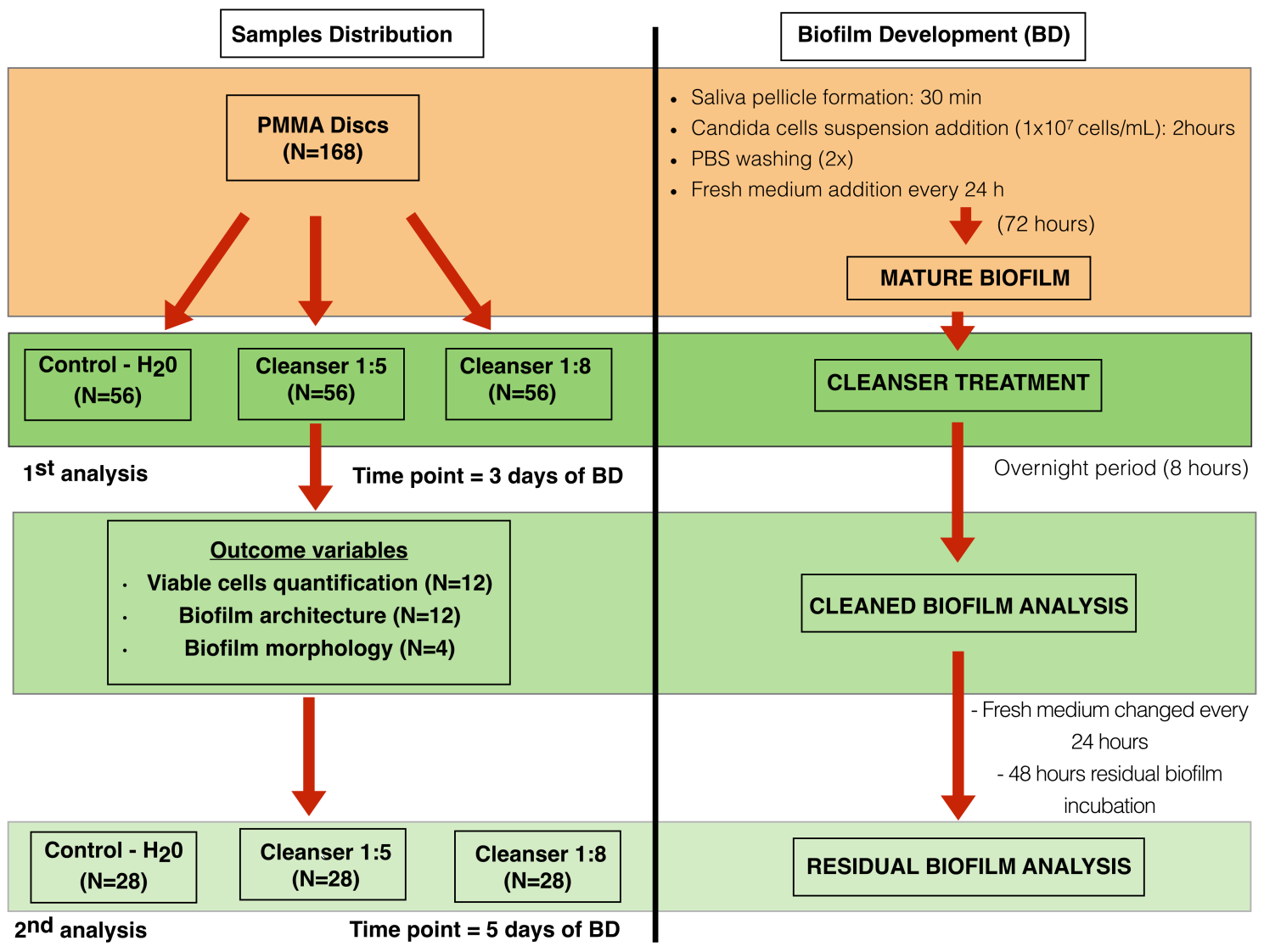

Supplement: Additional file 1 — Scheme of the experimental design performed in the present study. [file 1472-6831-14-77-S1.pdf]
